# Mountain glaciation drives rapid oxidation of rock-bound organic carbon

**DOI:** 10.1126/sciadv.1701107

**Published:** 2017-10-04

**Authors:** Kate Horan, Robert G. Hilton, David Selby, Chris J. Ottley, Darren R. Gröcke, Murray Hicks, Kevin W. Burton

**Affiliations:** 1 Department of Earth Sciences, Durham University, Durham DH1 3LE, UK.; 2 Department of Geography, Durham University, Durham DH1 3LE, UK.; 3 National Institute of Water and Atmospheric Research, Christchurch, New Zealand.

## Abstract

Over millions of years, the oxidation of organic carbon contained within sedimentary rocks is one of the main sources of carbon dioxide to the atmosphere, yet the controls on this emission remain poorly constrained. We use rhenium to track the oxidation of rock-bound organic carbon in the mountain watersheds of New Zealand, where high rates of physical erosion expose rocks to chemical weathering. Oxidative weathering fluxes are two to three times higher in watersheds dominated by valley glaciers and exposed to frost shattering processes, compared to those with less glacial cover; a feature that we also observe in mountain watersheds globally. Consequently, we show that mountain glaciation can result in an atmospheric carbon dioxide source during weathering and erosion, as fresh minerals are exposed for weathering in an environment with high oxygen availability. This provides a counter mechanism against global cooling over geological time scales.

## Teaser

Mountain glaciation greatly enhances oxidative weathering rates of organic carbon in rocks and associated carbon dioxide release.

## INTRODUCTION

The exposure of organic matter in rocks to oxidative weathering at Earth’s surface releases carbon dioxide (CO_2_) to the atmosphere from long-term (>10^6^ years) storage in the lithosphere and consumes atmospheric oxygen (O_2_) (*1*–*3*). The global CO_2_ emissions from the oxidation of rock-derived organic carbon [petrogenic OC (OC_petro_)] are estimated to be 40 to 100 × 10^6^ metric tons of carbon (tC) per year (*1*). Over million-year (geological) time scales, this emission represents the main source of atmospheric CO_2_ alongside volcanism and metamorphism (*1*–*4*) and plays a role in setting atmospheric O_2_ concentrations (*2*, *5*). Geological CO_2_ emissions are removed from the atmosphere by chemical weathering of silicate minerals by carbonic acid coupled to carbonate precipitation (*4*, *6*) and the burial of recently photosynthesized OC (*3*, *7*). These atmospheric CO_2_ drawdown mechanisms are regulated by erosion, temperature, and runoff and are thought to stabilize CO_2_ concentrations and global climate (*6*). However, we have little understanding of the factors controlling OC_petro_ oxidation rate (*1*, *8*, *9*) and hence how millennial-scale changes in climate (*10*) might modify this major CO_2_ emission.

Chemical weathering of OC_petro_ proceeds as surficial gases and fluids permeate through sedimentary rocks, oxidizing organic matter and releasing CO_2_ (*1*). The kinetics of OC_petro_ weathering appear to be ~10 times faster than the kinetics of silicate mineral weathering (*11*). Faster reaction kinetics imply that shorter fluid residence times are required to reach chemical equilibrium and maximize weathering fluxes during OC_petro_ oxidation (*6*), compared to acid hydrolysis silicate weathering (*6*, *12*). When considered together with the high concentrations of O_2_ in the present-day atmosphere, weathering models suggest that the OC_petro_ oxidation rate is set by the mineral supply rate in most locations globally (*13*). Microorganisms may also be important facilitators of OC_petro_ oxidation (*14*). On the basis of these observations, we propose that mountain glaciation could significantly enhance OC_petro_ oxidation rates due to a combination of physical and biogeochemical factors: (i) frost cracking and abrasive glacial grinding processes, which produce fine sediment with more surface area in an environment with high water availability (*15*, *16*); (ii) lower vegetation and soil cover that can increase the availability of O_2_ to exposed bedrock and in deeper soil horizons; and (iii) the activity of microorganisms catalyzing weathering, both subglacially and during primary ecological succession on moraines (*14*, *17*–*19*). Previous work has suggested that sulfide oxidation is enhanced subglacially (*18*, *20*). If OC_petro_ oxidation rates also increase, CO_2_ release may be highest during periods of repeated mountain glaciation over millennia (*21*) and may provide a mechanism for countering cooling trends in Earth’s climate over time scales of 100 thousand years to millions of years.

**Fig. 1:**
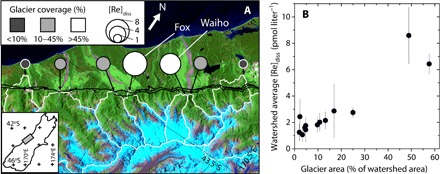
The western Southern Alps, New Zealand. (**A**) Watersheds for the central part of the study area where glacier area varies most, with watershed boundaries marked in white. The black line is the Alpine Fault trace. Image is from Landsat ETM (Enhanced Thematic Mapper) (31 December 2002), shown to illustrate glacial coverage. The watershed-averaged dissolved Re concentration, [Re]_diss_ (pmol liter^−1^), is shown as the circle size, and the shading reflects the percentage watershed area with glaciers (*23*). Inset shows the location of the study area on South Island, New Zealand. (**B**) Positive relationship between watershed-averaged [Re]_diss_ and the percentage of the watershed area covered by glaciers (*n* = 13, *r* = 0.93, *r*^2^ = 0.87, *P* < 0.001). Gray whiskers are ±2 SE on the mean [Re]_diss_ values.

Here, we examine the potential for mountain glaciation to increase OC_petro_ oxidation rates. We focus on the mountain watersheds of the western Southern Alps, New Zealand (Fig. 1A), where lithological contrasts are relatively small along strike of the Alpine Fault (*22*), but glacial coverage is variable (*23*). Previous work has suggested that high silicate weathering rates (*20*, *24*) are facilitated by rapid soil production (*25*) and mineral supply by bedrock landslides (*26*). Here, we assess the rates of oxidative weathering and the role of mountain glaciers using river water and sediment samples from 13 watersheds (see Materials and Methods). We also collected samples from two watersheds in the eastern Southern Alps that host glaciers but have lower physical erosion rates and from the Waipaoa River in North Island, which has a high erosion rate but no glaciers (*24*). In addition, we compile measurements from mountain watersheds draining OC_petro_-bearing sedimentary rocks in North America (Yukon and Mackenzie) and Asia (Taiwan rivers, Ganges, and Brahmaputra) (*9*, *27*).

To assess and quantify OC_petro_ oxidation rates, we measured the concentration of the redox-sensitive trace element rhenium (Re) in river waters ([Re]_diss_), river bed materials ([Re]_BM_), weathered colluvium and surface soils on hillslopes ([Re]_COL_) using isotope dilution and inductively coupled plasma mass spectrometry (ICP-MS) (see Materials and Methods). The close association of Re and OC_petro_ in sedimentary rocks (*28*) and the solubility of Re upon oxidation during weathering (*29*) (present as the soluble perrhenate oxyanion, ReO_4_^−^, in soils and rivers with pH values between 5.5 and 9.5) have led previous studies to suggest that Re can trace OC_petro_ oxidation (*1*, *9*, *30*, *31*). For river watersheds with similar runoff and bedrock composition, the dissolved Re concentration, [Re]_diss_, has been shown to reflect the relative rate of oxidative weathering (*9*). The dissolved Re discharge (mol year^−1^) or dissolved Re yield (mol km^−2^ year^−1^) provides a more direct means to quantify the oxidative weathering yield (*1*, *9*, *30*, *31*). The dissolved Re yield has been used to estimate the associated CO_2_ emissions by OC_petro_ oxidation when the Re to OC_petro_ ratio of the rocks undergoing weathering has been characterized (*9*). The main uncertainties in the use of the Re proxy derive from the following: (i) As a soluble element, Re may be mobilized more effectively during weathering than the CO_2_ derived from OC_petro_ oxidation (*30*); (ii) Re may be hosted in silicate and sulfide minerals (*27*); and (iii) graphitic OC_petro_ is less susceptible to oxidation (*32*).

## RESULTS

### River bed materials and weathered colluvium

The mean organic carbon (OC) weight percentage in river bed materials, [OC]_BM_, from the western Southern Alps is 0.13 ± 0.01 weight % (wt %) (*n* = 31, ±2 SE), and the mean stable carbon isotope composition is δ^13^C = −22.5 ± 0.6‰ (*n* = 31, ±2 SE). For comparison, the [OC]_BM_ values are lower than those in Taiwan (*9*) but more than double those measured in the Himalaya (*32*). The mean values of river bed materials are similar to previous measurements from this location (*22*, *33*) and to the mean of bedrock values in the western Southern Alps, [OC] = 0.15 ± 0.05 wt % and δ^13^C = −21.1 ± 1.1‰ (*n* = 11, ±2 SE) (*22*), suggesting that they are dominated by OC_petro_, with minor inputs from biospheric OC generated by recent photosynthesis by C_3_ plants (δ^13^C ~ −28‰). The river bed materials have less variability in their average composition when compared to bedrock, which likely reflects the integration of OC_petro_-bearing sediment from landslides and mass wasting processes, which can erode OC_petro_ from large areas of the watershed (*34*, *35*). The river sediments downstream are a mixture of these inputs (*22*). Although the river bed materials have a slightly lower OC concentration than bedrock in the mountain belt, they are indistinguishable within the variability in the means and can therefore provide a robust method to assess the watershed-averaged bedrock composition (*22*, *32*, *36*). This is consistent with findings in other erosive settings where bedrock landslides excavate deep into the landscape, tap into unweathered rock, and supply it to rivers in the sand-silt-clay fraction of river sediments (*37*).

The mean Re concentration in river bed materials, [Re]_BM_, is 118 ± 21 parts per trillion (ppt) (*n* = 31, ±2 SE) (table S1). Although this Re concentration is low for sedimentary rocks (*38*), it is in line with the low [OC]_BM_ in the western Southern Alps and consistent with organic matter being a dominant host of Re in sediments (*28*). The mean [Re]_BM_/[OC]_BM_ ratio is 0.9 ± 0.2 × 10^−7^ g g^−1^ (*n* = 31, ±2 SE), and there is no systematic variability along strike of the Alpine Fault (that is, no variability with latitude and longitude).

We examine samples of weathered colluvium collected from hillslopes in the western Southern Alps for loss of OC_petro_ and Re (table S2). In the Docherty Creek watershed, which neighbors the Waiho River, weathered colluvium has a mean [Re]_COL_ = 29 ± 6 ppt (*n* = 4, ±2 SE), compared to local river bed materials downstream at Docherty Creek (NZ14-90; [Re]_BM_ = 112 ppt). Soil litter samples are also depleted in Re (table S2). This is expected if Re loss occurs during oxidative weathering. The mean OC content of the colluvium, [OC]_COL_ = 1.2 ± 0.3 wt % (*n* = 4, ±2 SE), is higher than that of the local river bed materials (NZ14-90; [OC]_BM_ = 0.21 wt %). However, organic matter in the colluvium has a stable isotopic composition (mean δ^13^C = −25.9 ± 0.4‰) and radiocarbon activity (reported as fraction modern, *F*_mod_ = 0.80 ± 0.09) that are very different from the expected rock inputs (NZ14-90; δ^13^C = −21.2‰ and expected *F*_mod_ ~ 0). The values suggest an important contribution from biospheric OC in these samples (*22*).

To assess the OC_petro_ content of the colluvium and to account for biospheric OC, we use δ^13^C and *F*_mod_ values in a mixing analysis (see Materials and Methods). The high [OC]_COL_ of these samples and the relatively low OC_petro_ content of rocks in this mountain range (*22*), together with the observation that century-aged biospheric OC is important in these New Zealand soils (fig. S1), result in uncertainty on the absolute OC_petro_ contents. The mean [OC_petro_] of the weathered colluvium is calculated as 0.15 ± 0.06% (*n* = 4, ±2 SE), which is lower than that of the river bed material. The data are consistent with OC_petro_ loss during weathering on hillslopes. The coupled loss of Re and OC_petro_ during chemical weathering supports previous measurements on soil from the Ohio Shale (table S2) (*30*), the Himalaya (*39*), and Taiwan (*9*). The weathered colluvium from New Zealand and published soil data show that Re is generally more mobile during OC_petro_ weathering, and so we account for this when estimating the OC_petro_ weathering rate using the dissolved Re flux (see Materials and Methods).

### River waters

The major dissolved ions in rivers draining the western Southern Alps (Ca^2+^, Mg^2+^, Na^+^, and K^+^) characterize the overall weathering processes and reflect a source rock comprising metasedimentary silicate rocks hosting trace carbonate minerals (fig. S2A). The water measurements from this study in 2014 are consistent with sampling campaigns in 1998, 1999, and 2000 (*20*, *24*). Notably, all sampling campaigns find higher relative HCO_3_^−^ and Ca^2+^ concentrations in the heavily glaciated Fox and Waiho rivers (fig. S2A), which probably reflects the higher susceptibility of carbonate minerals to acid hydrolysis reactions in these watersheds. The overlap of the data sets collected from different years, seasons, and flow regimes (fig. S2, A and B) suggests that spatial patterns in dissolved ion composition are retained despite the potential for seasonal and flood-event scale variability (*40*).

The [Re]_diss_ values in western Southern Alps rivers range from 0.81 to 11.55 pmol liter^−1^, with a mean = 3.05 ± 0.69 pmol liter^−1^ (*n* = 51, ±2 SE) (table S3). There is a distinct variability between different watersheds (Fig. 1A), with the Waiho and Fox watersheds having the highest mean [Re]_diss_ values throughout the sampling period. Mean [Re]_diss_ is not correlated with [Re]_BM_ in the western Southern Alps, suggesting that the bedrock geology does not set the spatial pattern in [Re]_diss_. When all the data are considered together, the watershed-averaged [Re]_diss_ is correlated with the proportion of area covered by glaciers upstream (Fig. 1B; *r*^2^ = 0.87, *P* < 0.001, *n* = 13).

The [Re]_diss_ values are generally low compared to those measured in river waters globally (*27*) and in rivers draining metasedimentary rocks in Taiwan, which have values ranging from ~5 to 25 pmol liter^−1^ (*9*). However, when [Re]_diss_ values are normalized to the concentration of Re in river bed materials, [Re]_BM_ (*9*, *32*, *36*), the values are more similar to those in Taiwan. In the western Southern Alps, watersheds dominated by river erosion and bedrock landslides (that is, not by glacial erosion processes) have a mean [Re]_diss_ = 1.92 ± 0.76 pmol liter^−1^ and mean [Re]_BM_ = 105 ± 20 ppt, giving a [Re]_diss_/[Re]_BM_ = 3.4 ± 1.4 × 10^−3^ (pg g^−1^/pg g^−1^), which is slightly higher than that measured in Taiwan, where the average [Re]_diss_/[Re]_BM_ is 3.3 ± 0.5 × 10^−3^. The two mountain belts have comparable physical erosion rates (*9*, *24*), suggesting that Re mobility is similar between sites despite the contrasts in bedrock geology. The [Re]_diss_/[Re]_BM_ values are more than double in the Waiho and Fox watersheds that host valley glaciers and have extensive glacial coverage.

To estimate oxidative weathering yields, we quantify the dissolved Re yield (mol km^−2^ year^−1^) in watersheds where we have annual water discharge estimates (*41*). These are in the Hokitika, Whataroa, Haast, and Waiho rivers (tables S4 and S5). Our river water samples from 2014 cover a relatively narrow dynamic range in water flow especially in the glacial watershed (~0.5 to 1.5 times mean flow values) but do not show significant dilution at a higher flow (fig. S2C and table S5). This suggests that the annual water discharge (*Q*_annual_, m^3^ year^−1^) (*41*) and our mean [Re]_diss_ for each large watershed can together provide a reliable estimate of dissolved Re flux. However, the average ion concentrations collected at lower *Q*_w_ may overestimate the dissolved ion flux, if ions are diluted at a high flow (*40*). We find that using an average concentration does not systematically overestimate or underestimate the dissolved Re yield (see Materials and Methods). To assess the role of seasonal and/or interannual variability, we plot our major ion data (for example, Ca^2+^) alongside daily water discharge (*Q*_w_, m^3^ s^−1^) for the Whataroa and Hokitika watersheds (fig. S1B) and compare this to published data from 1998 to 1999 (*24*) and 2000 to 2001 (fig. S2B) (*20*). The broad consistency in ion concentrations and ion ratios suggest that annual and seasonal variability may be of second-order importance when compared to contrasts between different watersheds. Rainwater and hydrothermal water samples (table S3) have very low [Re]_diss_ (<0.2 pmol liter^−1^), so no correction is made to the Re flux from these inputs.

### Global compilation

**Fig. 2:**
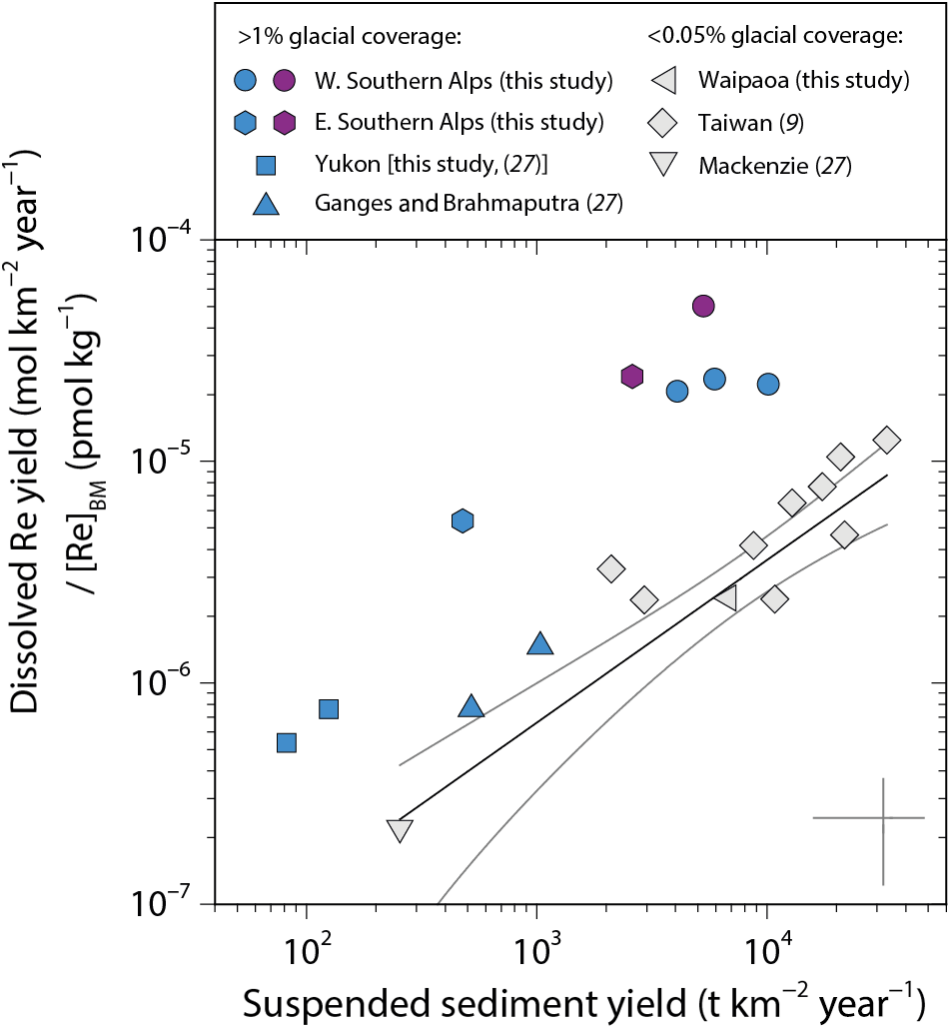
Dissolved Re yield in mountain watersheds around the world draining sedimentary rocks as a function of suspended sediment yield. Dissolved Re yields (mol km^−2^ year^−1^) have been normalized to river bed material Re concentration ([Re]_BM_, pmol kg^−1^) to account for lithological variability between watersheds (table S6). Gray whiskers show ±50% of the values. Gray symbols represent watersheds with <0.05% of their area covered by glaciers, with the power law best fit to data shown by the black line and the 95% confidence intervals shown in gray [*y* = (4.1 ± 3.4 × 10^−9^)*x*^(0.7 ± 0.1)^, *r*^2^ = 0.82, *P* < 0.001, *n* = 12]. Blue symbols represent watersheds with glaciers covering >1% of the watershed area, and purple symbols represent watersheds with the highest coverage of glaciers (>40%).

Published data are compiled from watersheds around the world that allow for a comparison to our findings in New Zealand (table S6). The required data are as follows: (i) dissolved Re concentration ([Re]_diss_); (ii) watershed-averaged bedrock Re composition, an indication of which is provided by [Re]_BM_; (iii) annual water discharge to estimate dissolved Re yield; and (iv) suspended sediment yield as a proxy of physical erosion rate (*9*, *20*, *22*, *27*, *41*–*45*). We also add new measurements of river water and bed material samples from the Jollie and Hooker watersheds draining the eastern Southern Alps, the Waipaoa River in North Island, New Zealand, and the Yukon River and Mackenzie River in Canada, which we collected using similar methods. We estimate the dissolved Re yield for each of these sites and account for variability in the bedrock geology by normalizing the Re yield to the measured [Re]_BM_. We quantify the upstream area covered by glaciers from published work or by using the World Glacier Inventory as we do for the western Southern Alps (see Materials and Methods). We find dissolved Re yield (normalized by [Re]_BM_) increases with increasing annual suspended sediment yields across the data set (Fig. 2), supporting previous work from Taiwan (*9*). However, watersheds with mountain glaciation upstream of the sampling locations (where glaciers cover >1% of the area) have a higher dissolved Re yield for a given suspended sediment yield (Fig. 2).

## DISCUSSION

The erosion rates in the western Southern Alps are high (4000 to 10,000 t km^−2^ year^−1^) and comparable to those in Taiwan (*9*, *24*). The similarity in Re mobility between these settings agrees with the notion that high erosion rates can enhance oxidative weathering of OC_petro_ and the release of Re to the dissolved load of rivers (*9*). This probably reflects the rapid soil formation in the western Southern Alps (*25*) and is consistent with an important role of bedrock landslides for exposing bedrock clasts in landslide deposits and focusing hydrological pathways in landslide scars (*26*). In the global data compilation, we find that suspended sediment yield (a proxy for physical erosion rate) is correlated with dissolved Re yield (Fig. 2). This suggests that OC_petro_ oxidation is supply-limited in many locations, as predicted by the relatively fast kinetics of OC_petro_ oxidation and the high atmospheric O_2_ concentrations at present (*11*, *13*). This contrasts with acid hydrolysis weathering of silicate minerals, which is thought to be kinetically limited at the high erosion rates experienced in mountain belts (*6*, *12*). The implication is that for watersheds underlain by sedimentary rocks, increased erosion may result in a less effective CO_2_ drawdown by silicate weathering, whereas CO_2_ release by oxidative weathering of OC_petro_ continues to increase (Fig. 2).

In the western Southern Alps, we find that glacial cover is a major control on the average [Re]_diss_ measured in watersheds (Fig. 1B). This is not only the case for the watersheds with large valley glaciers (the Waiho and Fox; Fig. 1A): Glacial processes appear to enhance oxidative weathering to some degree in all watersheds. Although this may seem to conflict with the idea that OC_petro_ oxidation in mountains is already supply-limited (*9*), there are characteristics of glacial watersheds, such as (i) physical mechanisms that increase effective surface area (*15*, *16*) and (ii) biogeochemical mechanisms that increase O_2_ availability and the competitiveness of microbial communities (*17*), which mean that for the same physical erosion rate, oxidation rate may be further enhanced. Physical mechanisms include glacial abrasion (*16*), which can supply large quantities of fine material for weathering within the glacial system and in the deposited moraines. At higher elevations on steep rock walls, freeze/thaw cycles and frost cracking driven by sustained subzero temperatures and water availability in the porous bedrock can also increase the supply of fresh, fine material to O_2_ in the air and water (*15*). Biogeochemical factors work in parallel, with limited vegetation and soil development in glacial watersheds resulting in less demand for O_2_ by heterotrophic respiration (*17*). Oxygen could therefore penetrate deeper into exposed rock surfaces (*11*, *13*). Microbial communities also facilitate OC_petro_ oxidation (*1*, *14*) and are active both subglacially (*18*, *19*) and in moraines colonized by organisms during primary succession (*17*).

The mechanisms described here are not unique to watersheds of the western Southern Alps but should operate wherever mountain glaciation occurs on OC_petro_-bearing rocks. In the glaciated eastern Southern Alps watersheds, erosion rates are lower than those in the western Southern Alps (*15*, *20*, *24*), and so lower OC_petro_ oxidation rates may be expected (*9*). However, when compared to watersheds with similar erosion rates in unglaciated Taiwan, the glaciated watersheds have higher dissolved Re yields (Fig. 2). The glacier-free Waipaoa River data are consistent with the data from Taiwan. When we examine larger rivers draining OC_petro_-bearing sedimentary rocks (table S6), we find that a global pattern starts to emerge (Fig. 2). Erosion rate is a first-order control on oxidative weathering rate (dissolved Re yield), but watersheds hosting glaciers (>1% of the watershed area; for example, Yukon, Brahmaputra, Ganges, and Southern Alps) have dissolved Re yields that are up to three times greater for a given erosion rate than watersheds with very low glacial coverage, regardless of the basin area.

The dissolved Re yield can be used to estimate the CO_2_ oxidation yield, *J*_CO2_ (gC km^−2^ year^−1^), by OC_petro_ oxidation (*1*, *9*, *30*, *31*). First, there must be good constraint on the Re-to-OC ratio of the sedimentary rocks and the behavior of Re and OC_petro_ during weathering. In the western Southern Alps, the river bed materials provide an estimate of watershed-averaged [Re] and [OC_petro_], and their compositions are similar to measured bedrocks. The weathered colluvium confirms coupled Re and OC_petro_ loss (fig. S2 and table S2) (*1*, *9*, *31*). To quantify the CO_2_ release, the dissolved Re yield in grams (*J*_Re_, g km^−2^ year^−1^) is combined with the [OC]_BM_/[Re]_BM_ (g g^−1^) (Eq. 1).

$$J_{\text{CO}2}=J_{\text{Re}}\times ({\left[\text{OC}\right]}_{\text{BM}}/{\left[\text{Re}\right]}_{\text{BM}})\times f_{C}\times (1-f_{\text{graphite}})$$ (1)

We correct the estimated CO_2_ release to account for the relative mobility of Re and OC_petro_ during weathering in soils, with *f*_C_ being the ratio between percentage loss of OC_petro_ in soil versus percentage loss of Re in soil. This factor also accounts for the role of sulfide and silicate minerals as trace sources of dissolved Re (*9*, *27*). On the basis of the published data from soils (*9*, *30*, *39*) and our measurements from the western Southern Alps (table S3), *f*_C_ is expected to be <1 but >0.5. To account for the presence of graphite, which may not be oxidized, we vary the fraction of OC_petro_ as graphite (*f*_graphite_) from 0.5 to 0, informed by measurements from the study location (*33*). The CO_2_ oxidation flux, *J*_CO2_ (gC km^−2^ year^−1^), is calculated using a Monte Carlo simulation to account for these uncertainties (see Materials and Methods).

In the western Southern Alps, watersheds with limited glacial coverage are estimated to release 14 ^+9^/_−5_ tC km^−2^ year^−1^ (Whataroa) by OC_petro_ oxidation using the Re proxy (table S4). These are similar to OC_petro_ oxidation yields estimated in Taiwan where erosion rates are similar (*9*). This suggests that the Re proxy is producing consistent results at the watershed scale. The Re-derived estimate of CO_2_ flux from OC_petro_ oxidation for the glaciated Waiho watershed is approximately double, at 30 ^+20^/_−11_ tC km^−2^ year^−1^. These values are similar to seasonal measurements of soil respiration in a primary succession on sedimentary rocks exposed by recent glacial retreat in Svalbard (~10 to 24 tC km^−2^ year^−1^, based on monthly averaged data) (*46*) but lower than typical rates of soil respiration in mineral soils that contain non–rock-derived organic matter (*47*). Although the uncertainties on the CO_2_ fluxes are relatively large on the basis of our current understanding of Re and OC_petro_ mobility, the difference between the glaciated watersheds and the other watersheds is larger than these uncertainties (table S4).

**Fig. 3:**
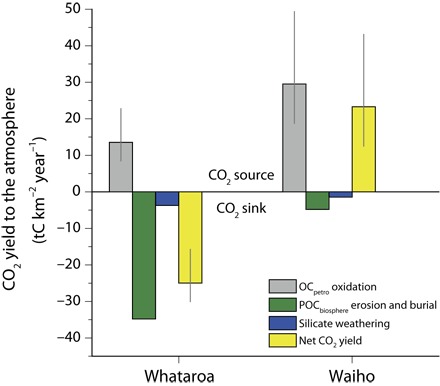
Net carbon balance due to erosion and weathering in the western Southern Alps. Two watersheds with contrasting glacial coverage area are shown: the Whataroa (9.7% glacier coverage) and the Waiho (57.6% glacier coverage). The CO_2_ release to the atmosphere by OC_petro_ weathering (this study; from dissolved Re measurements) is shown alongside the CO_2_ drawdown by erosion and burial of biospheric particulate OC (POC_biosphere_) (*22*) and silicate weathering (*20*, *24*).

In watersheds where mountain glaciers are confined to headwaters, the Re-derived estimates of CO_2_ release do not negate CO_2_ drawdown by silicate weathering [~2 to 10 tC km^−2^ year^−1^ (*24*)] and by erosion and sedimentary burial of biospheric OC [Fig. 3; ~40 tC km^−2^ year^−1^ (*22*)]. In stark contrast, the doubling of the OC_petro_ oxidation rate in the Waiho watershed converts it into a net CO_2_ source during erosion and weathering (Fig. 3). At present, the Waiho and Fox rivers drain less than 5% of the sampled area (Fig. 1A), so the enhanced glacial contribution to CO_2_ fluxes from OC_petro_ oxidation has a modest influence across the mountain belt. However, under more heavily glaciated conditions, the Southern Alps would be primed to act as a CO_2_ source. Accelerated OC_petro_ oxidation driven by the physical and biogeochemical mechanisms that we have identified may have increased CO_2_ emissions (Fig. 2). The heavily glaciated western Southern Alps watersheds indicate that biospheric OC erosion and burial will also decrease as glacial cover increases. In addition, at high erosion rates and high rates of mineral supply, silicate weathering rates are very sensitive to temperature and runoff (*6*, *12*), and cooler temperatures may decrease rates of CO_2_ drawdown (Fig. 3).

The global CO_2_ emissions by OC_petro_ oxidation remain to be better quantified. Nevertheless, they are probably as large as those from volcanic degassing (*1*). To evaluate the strength of this feedback in glaciated mountain belts for counteracting global cooling and for potentially having the capacity to end a glaciation, both the OC_petro_ presence and abundance in glaciated mountain ranges must be taken into account. By way of example, the Himalaya has the potential to be a key site for CO_2_ fluxes by OC_petro_ oxidation because it hosts OC_petro_-bearing Tethyan Sedimentary Series shales at high altitudes. Furthermore, around the world, rocks containing OC_petro_ have been subject to repeated mountain glaciation throughout the Late Cenozoic. Enhanced OC_petro_ oxidation in locations such as the Rockies, the Andes, and the European Alps (*21*) could have driven these sites to operate as important CO_2_ sources and O_2_ sinks during sustained periods of global cooling and glaciation over 10^4^ to >10^6^ years (*5*). OC_petro_ oxidation could act in tandem with transient CO_2_ emissions from enhanced sulfide oxidation and weathering of carbonate minerals by sulfuric acid, during glaciation (*48*). Although the global fluxes are difficult to quantify from the available data (Fig. 2), enhanced OC_petro_ oxidation associated with more extensive glacial erosion processes is consistent with the ~2% decline in atmospheric O_2_ recorded in ice cores over the past 800,000 years (*5*). If the decline in atmospheric O_2_ results from changes in OC burial versus OC_petro_ oxidation alone, it implies a net CO_2_ release of ~3 × 10^11^ mol year^−1^ or ~3 to 4 × 10^6^ tC year^−1^ (*5*). This corresponds to only a modest (~6%) increase in global OC_petro_ oxidation rates (*1*) over this period in which the duration and intensity of glaciation increased (*21*). We propose that the link between OC_petro_ weathering and mountain glaciation offers a previously unrecognized feedback between climate and the carbon cycle, where increased CO_2_ emissions act to counter further global cooling during the Late Cenozoic.

## MATERIALS AND METHODS

### Sample collection

Samples were collected from 13 watersheds in the western Southern Alps, which drain to the west of the main divide, two draining the eastern Southern Alps to the east of the main divide and the Waipaoa River in North Island, New Zealand (tables S1 to S3). Together, these watersheds allowed us to examine the dual roles of physical erosion rate (*9*) and glacial coverage on OC_petro_ weathering. The Southern Alps is a steep mountain belt built by transpression along the Alpine Fault. The western flank has a temperate climate, with a high erosion rate driven by orographic precipitation (exceeding 8 m year^−1^), steep slopes, and bedrock landslides, which expose OC_petro_ in metasedimentary rocks (*34*). Previous work has documented high rates of silicate and carbonate weathering in the western Southern Alps (*20*, *24*). Erosion dominates the denudation, with the largest percentage of chemical denudation occurring in the Haast watershed (5%) (*24*). Along the western Southern Alps, the metamorphic grade varies perpendicular to the Alpine Fault strike, but the sedimentary protolith is similar in all watersheds, and the OC_petro_ content ranges from ~0.1 to ~0.2% (*22*, *33*). In contrast, there are significant differences in glacial coverage (Fig. 1A), including two watersheds with large valley glaciers (Waiho and Fox) and some frontal watersheds with very minimal glacial coverage (for example, Waitangitaona). The eastern Southern Alps is also dominated by glacial processes but experiences lower precipitation (<2 m year^−1^), lower rates of bedrock landsliding (*15*), and slower long-term exhumation rates (*49*). As a result, the chemical denudation rates are also lower (*50*). The Waipaoa River in North Island, New Zealand drains lower metamorphic grade OC_petro_-bearing sedimentary rocks at high erosion rates but lacks glacial influence (*51*, *52*).

To assess OC_petro_ oxidation in the western Southern Alps, we sampled the dissolved products of chemical weathering, weathered colluvium on hillslopes, and relatively unweathered river bed materials of sand and finer grade. River waters (*n* = 51) were collected from the center of river channels at their surface. Watersheds were sampled two to six times over 1 month (14 September 2014 to 03 October 2014) at variable flow to examine the hydrological variability of the dissolved ions released from chemical weathering (*40*). Water samples were decanted to sterile plastic containers before filtration through 0.2-μm polyethersulfone filters with a diameter of 142 mm within a day of collection and stored in acid-cleaned low-density polyethylene bottles. Alkalinity measurements were made by Gran titration on an aliquot of filtered water. All water samples intended for cation and Re analysis were acidified in the field to pH ~2 (*9*, *31*), with an unacidified aliquot for anion analyses. Two 250-ml rainwater samples were collected over 10-hour periods (table S1).

In the western Southern Alps, soils are thin and weathering profiles are often poorly developed (*25*). Landslide-derived colluvium is an important locus of weathering (*26*). Therefore, to characterize Re and OC_petro_ behavior in the weathering zone, we collected ~500-cm^3^-sized bulk samples of weathered colluvium at discrete depths between 10 and 70 cm below the soil surface at three sites on the forested hillslopes of Alex Knob, which drains to the Docherty Creek watershed. We also collected surface soil samples from the upper 3 cm that comprised a mixture of litter and mineral soil (*n* = 5) using a metal trowel and transferred samples to sterile plastic bags (table S3).

River bed material samples (*n* = 31) were also collected to help constrain the composition of unweathered materials (tables S2 and S3) (*9*). Samples were taken from channel edges or bank deposits that were taken to represent the sand-to-silt fraction deposited on the river bed during recent flow regimes and transferred to sterile plastic bags. River water samples and bed materials were also collected from the eastern Southern Alps and Waipaoa River using these methods (table S2).

### Geochemical analyses

Dissolved Re concentrations in river water samples ([Re]_diss_, pmol liter^−1^ ) were measured by isotope dilution quadrupole ICP-MS (Q-ICP-MS) in conjunction with anion exchange column chemistry to preconcentrate and purify Re. Between 30 and 500 ml of water samples was doped with a known amount of tracer solution consisting of enriched ^185^Re and evaporated to dryness, with the dried sample dissolved in HNO_3_ before anion exchange column chemistry.

Solid samples (surface soil, weathered colluvium, and river bed materials) were ground to a fine powder before acid digestion to generate an integrated bulk sample. Homogenizing bedload samples permitted assessment of the average Re composition of the rocks at the watershed scale for each river because fluvial transit times are short. A known weight of powder (~0.5 g) was doped with a known amount of ^185^Re spike and digested in a 6:3 HF-HNO_3_ mix (9 ml) for 24 hours at 120°C and then evaporated. The dried sample was further digested in a 2:1 mix of HNO_3_-HCl (3 ml) for 24 hours at 120°C and then evaporated. Re was isolated and purified using a NaOH-acetone solvent extraction methodology (*53*).

The Re isotope composition of the purified Re aliquots were determined in a 0.8 N HNO_3_ medium using a Thermo Fisher Scientific X-Series Q-ICP-MS at Durham University. The procedural blank was ~1% of the lowest concentration samples. Uncertainties in the Re abundance were determined by error propagation of uncertainties in Re mass spectrometry measurements, blank abundance and isotopic compositions, spike calibrations, and reproducibility of standard Re isotopic values. Repeat analyses of [Re]_diss_ in a river water standard, SLRS-5, gave a concentration of 59.8 ± 1.7 ppt (*n* = 12, ±2 SE), in agreement with the previously reported value of 66 ± 12 ppt (*54*).

Major ion concentrations in water samples were analyzed by ion chromatography. Cation and anion standards and a certified reference standard (LETHBRIDGE-03) were run to validate the analytical results. The HCO_3_^−^ concentration was estimated using total alkalinity, temperature, and pH (measured in the field) data inputted to CO2SYS (*55*). The charge balance of dissolved cations (TZ^+^ = Na^+^ + K^+^, 2Mg^2+^ + 2Ca^2+^) and dissolved anions (TZ^−^ = Cl^−^ + HCO_3_^−^ + 2SO_4_^2−^) was determined ([TZ^+^ − TZ^−^]/[TZ^+^ + TZ^−^]) as a measure of data quality (*20*). This was 11% across all samples, within the combined uncertainty and similar to previous work in this location (*20*).

In river bed materials, surface soils, and weathered colluvium, the OC concentration ([OC], %) was measured following a 0.2 N HCl leach protocol (*56*), which was tested on samples from this location to ensure full removal of detrital carbonates. Aliquots of samples were combusted, and the concentration and stable isotope composition of OC (δ^13^C, ‰) were determined using a Costech elemental analyzer coupled to a Thermo Fisher Scientific Delta V Advantage isotope ratio mass spectrometer at Durham University. Corrections for procedural and instrument blanks were applied, and the result normalized to the composition of international standards (reported relative to Vienna Pee Dee Belemnite with a precision of 0.2‰). The radiocarbon activity (reported as the fraction modern, *F*_mod_) was measured on four colluvium samples and two soil litters by accelerator mass spectrometry at the University of California, Irvine Keck Carbon Cycle facility, following graphitization. Sample preparation background was subtracted based on measurements of ^14^C-free coal processed through the full protocol (table S3).

### OC_petro_ content of weathered colluvium

To assess the OC_petro_ content of the weathered colluvium samples and to account for OC derived from recent productivity (biospheric OC), we adopted a mixing analysis based on observations of δ^13^C and *F*_mod_ values. The ^14^C activity of sedimentary rocks is generally considered to be below the analytical background, that is, *F*_mod_ = 0, and thus distinct from modern biospheric OC (*F*_mod_ ~ 1) and degraded soil of ~1000 years old (*F*_mod_ ~ 0.9). The δ^13^C of OC_petro_ in the Southern Alps was ^13^C-enriched (δ^13^C ~ −21 to −22‰) compared to the terrestrial biosphere (dominated by C_3_ plants) (*22*).

The stable isotope composition of an element shown against the reciprocal of its concentration can reveal mixing trends or processes that alter the concentration and fractionate isotopes. Surface soil litters are OC-rich and ^13^C-depleted, and they describe a linear trend between δ^13^C_org_ and 1/[OC], albeit one that only describes ~40% of the variability in the data. This is consistent with the degradation of plant-derived OC in surface soils, loss of OC, and enrichment in ^13^C (fig. S2A). In contrast, the weathered colluvium samples define a different linear trend, which we interpret as a mixture of degraded biospheric organic matter (originally derived from the surface soil) with ^13^C-enriched, OC-poor material from the sedimentary rocks (fig. S2B). The intercept of these two trends implies that the degraded soil OC has a value of −26.8 ± 0.8‰ (propagating the 95% uncertainty bounds on the linear trends).

The weathered colluvium samples are also ^14^C-depleted (fig. S2B), which is consistent with OC_petro_ addition. The samples can be described by a linear trend between *F*_mod_ and δ^13^C_org_ that intercepts biospheric and petrogenic OC (fig. 2B). Using the “degraded soil” δ^13^C_org_ value (fig. S2A) and the trend defined by the samples, we estimated the *F*_mod_ of the biospheric OC in these samples as *F*_mod_ = 0.93 ± 0.36. These values and their uncertainties were used in a two-component end member mixing model (*51*) to quantify the fraction of OC_petro_ (*f*_petro_) and the corresponding [OC]_petro_ (*f*_petro_ × [OC]) (table S3). This approach does not consider additional aging of biospheric OC, which would act to reduce the *F*_mod_ of the biospheric OC. We therefore calculated an upper bound on the [OC_petro_] and a lower bound on the loss of OC_petro_ during weathering. The weathered colluvium samples had an average [OC_petro_] = 0.15 ± 0.06% (*n* = 4, ±2 SE), which is lower than the local river bed materials in this watershed ([OC]_BM_ = 0.21%) (table S3). Coupled loss of Re and OC_petro_ during chemical weathering supports measurements on soils from the Ohio Shale (table S1) (*30*), the Himalaya (*39*), and Taiwan (*9*). These data sets generally show that Re is more mobile during weathering, and so the dissolved Re flux must be corrected when estimating an OC_petro_ weathering rate (*9*).

### Dissolved Re flux

To assess whether the average of the ion concentrations collected at low *Q*_w_ may overestimate the dissolved ion flux, we compared the flux calculated using an average concentration method to that calculated by a rating curve method (taking into account dilution) using published data sets from the study location (*24*). For these data, the average measured [Ca^2+^] multiplied by the mean annual runoff returns a Ca^2+^ flux of 4.21 × 10^8^ mol year^−1^ for the Hokitika River. If we model the [Ca^2+^]-*Q*_w_ relationship (fig. S1B) as a power law rating curve (*57*) and apply it to the daily *Q*_w_ data from 1971 to 2015, the annual Ca^2+^ flux = 4.39 × 10^8^ mol year^−1^. The methods agree within 4%, with the average concentration method slightly underestimating flux. In comparison, for the SO_4_^2−^ flux, which also shows dilution with *Q*_w_ in published data (*24*), the difference in estimated fluxes is 3%. Assuming similar dilution trends for [Re]_diss_ as for [Ca^2+^] and [SO_4_^2−^], which is suggested on the basis of the available data (fig. S2C), results in a <5% underestimation of flux. Although a longer time series sampling would be informative for tracking the dissolved ion source and linking it to hydrological pathways (*40*, *57*), an average concentration with an accuracy of within ~5% is adequate for calculating the flux.

### Quantification of OC_petro_ oxidation rate and its uncertainty

To convert the dissolved Re flux to an estimate of CO_2_ release, there are known uncertainties related to the behavior of Re and OC_petro_ during weathering (*1*, *9*). The main uncertainties in this setting are as follows: (i) Because Re is a soluble element, oxidative weathering may mobilize Re more effectively during early soil formation, meaning that some Re measured in river waters does not correspond to release of CO_2_ at the weathering site (*30*); (ii), partly related to (i), some dissolved Re may come from sulfide and silicate minerals (*9*, *27*); and (iii) graphitic OC_petro_ may not be susceptible to oxidation and remain in the soil (*32*, *58*). Regarding point (i) and (ii), soils from Taiwan suggest congruent dissolution of Re, and loss of OC_petro_ can occur during weathering of young, thin soils (*9*). On a soil profile developed on OC_petro_-rich shale from Ohio (*30*), Re depletion reaches 100% in the most highly weathered soil, whereas OC_petro_ loss is ~70% (table S1). The data from weathered colluvium in the western Southern Alps are more consistent with the Ohio Shale than Taiwan. For point (iii), graphite is present in rocks within a kilometer of the Alpine Fault, making up almost 50% of the OC (*33*), and its measured abundance decreases to small amounts ~10 to 20 km from the Alpine Fault (*59*).

To account for and quantify the uncertainties on the CO_2_ flux estimate we used a Monte Carlo simulation. This includes uncertainty on the dissolved Re flux, the measured variability in [OC]_BM_/[Re]_BM_, and the assumptions (i), (ii), and (ii) above. For each watershed (area = A km^2^), we used ±2 SE on the mean for the [Re]_diss_ and the [OC]_BM_/[Re]_BM_ ratio. To account for the relative mobility of Re and OC_petro_ during weathering (*30*), we defined *f*_C_ as the ratio between the percentage loss of OC_petro_ in soil compared to bedrock and the percentage loss of Re in soil compared to bedrock. On the basis of published work, we varied *f*_C_ from 0.5 to 1 (*9*, *30*). To account for the presence of graphite, which may not be oxidized, we varied the fraction of OC_petro_ as graphite (*f*_graphite_) from 0.5 to 0, informed by measurements from the study location (*33*). The CO_2_ oxidation flux, *J*_CO2_ (gC km^−2^ year^−1^), was then calculated using Eq. 1.

The Monte Carlo simulation was run 100,000 times for each watershed, with a “full probability” distribution for each variable. We reported the median value ± 1 SD range. This reflects the present state of knowledge of the Re proxy for OC_petro_ oxidation. Future work should seek to refine this approach and reduce the uncertainties, for a better understanding of *f*_C_ and *f*_graphite_. By doing so, it may be possible to quantify watershed-scale fluxes using Re measurements more widely (*1*).

### Quantification of glacier cover using the World Glacier Inventory

The relative importance of glacial processes in the watersheds of the western Southern Alps was quantified using glacier locations and areas from the World Glacier Inventory (*23*). The total sum of glacier area (km^2^) was quantified for each watershed using ArcGIS, with flow routing algorithms to isolate drainage areas (table S4). In the global data set, published estimates were used (table S6).

### Biospheric OC erosion and burial

Previous work has estimated the erosion rate of biospheric OC in watersheds of the western Southern Alps (*22*). To estimate how this contributes to CO_2_ drawdown, we required an estimate of the burial efficiency of OC. On the basis of a recent global compilation (*44*), the sediment yield to an offshore basin plays an important role in setting the burial efficiency of OC. This is because sediment accumulation rate is a first-order control on OC burial efficiency in many marine environments (*60*). Using the sediment yield of ~6000 to 10,000 t km^−2^ year^−1^ for the western Southern Alps (*22*), the burial efficiency would be predicted to be from ~40% to 100% based on the global data set (*44*). A high burial efficiency would be consistent with the very high preservation potential of terrestrial palynomorphs offshore from the western Southern Alps (*61*). To provide a conservative estimate of biospheric OC burial, we used the lowest value in this range (40%) and multiplied the erosional export flux (*22*) by the OC burial efficiency to estimate CO_2_ drawdown by biospheric OC erosion as shown in Fig. 3.

## Supplementary Material

http://advances.sciencemag.org/cgi/content/full/3/10/e1701107/DC1

## SUPPLEMENTARY MATERIALS

Supplementary material for this article is available at http://advances.sciencemag.org/cgi/content/full/3/10/e1701107/DC1 fig. S1. Weathered colluvium from the western Southern Alps. fig. S2. Dissolved major ion concentrations in the western Southern Alps. table S1. River bed materials. table S2. Re and OC_petro_ in weathered colluvium. table S3. Major ion and Re concentration data for water samples from the Southern Alps, New Zealand. table S4. Western Southern Alps watershed average data and dissolved Re yield estimates. table S5. Hydrological data for watersheds with river gauging stations. table S6. Global watershed averaged Re measurements from mountain rivers draining sedimentary rocks. Reference (*62*–*66*)
