# Supplementary material for: Mountain glaciation drives rapid oxidation of rock-bound organic carbon
Source: Sci Adv. 2017 Oct 4;3(10):e1701107. doi: 10.1126/sciadv.1701107 (PMC5627982; doi:10.1126/sciadv.1701107)
Supplement: http://advances.sciencemag.org/cgi/content/full/3/10/e1701107/DC1 [file supp_3_10_e1701107__index.html]

Science Advances | Science Advances

## Supplementary Materials

**This PDF file includes:**

- fig. S1. Weathered colluvium from the western Southern Alps.
- fig. S2. Dissolved major ion concentrations in the western Southern Alps.
- table S1. River bed materials.
- table S2. Re and OCpetro in weathered colluvium.
- table S3. Major ion and Re concentration data for water samples from the Southern Alps, New Zealand.
- table S4. Western Southern Alps watershed average data and dissolved Re yield estimates.
- table S5. Hydrological data for watersheds with river gauging stations.
- table S6. Global watershed averaged Re measurements from mountain rivers draining sedimentary rocks.
- Reference (*62–66*)

Download PDF

**Files in this Data Supplement:**

- Adobe PDF - 1701107\_SM.pdf
