# Supplementary material for: Mountain glaciation drives rapid oxidation of rock-bound organic carbon
Source: Sci Adv. 2017 Oct 4;3(10):e1701107. doi: 10.1126/sciadv.1701107 (PMC5627982; doi:10.1126/sciadv.1701107)
Supplement: http://advances.sciencemag.org/cgi/content/full/3/10/e1701107/DC1 [file 1701107_SM.pdf]

Science Advances

AAAS

advances.sciencemag.org/cgi/content/full/3/10/e1701107/DC1

Supplementary Materials for

Mountain glaciation drives rapid oxidation of rock-bound organic carbon

Kate Horan, Robert G. Hilton, David Selby, Chris J. Ottley, Darren R. Gröcke, Murray Hicks, Kevin W. Burton

Published 4 October 2017, *Sci. Adv.* **3**, e1701107 (2017)
DOI: 10.1126/sciadv.1701107

This PDF file includes:

- fig. S1. Weathered colluvium from the western Southern Alps.
- fig. S2. Dissolved major ion concentrations in the western Southern Alps.
- table S1. River bed materials.
- table S2. Re and OC_{petro} in weathered colluvium.
- table S3. Major ion and Re concentration data for water samples from the Southern Alps, New Zealand.
- table S4. Western Southern Alps watershed average data and dissolved Re yield estimates.
- table S5. Hydrological data for watersheds with river gauging stations.
- table S6. Global watershed averaged Re measurements from mountain rivers draining sedimentary rocks.
- Reference (*62–66*)

Supplementary Materials

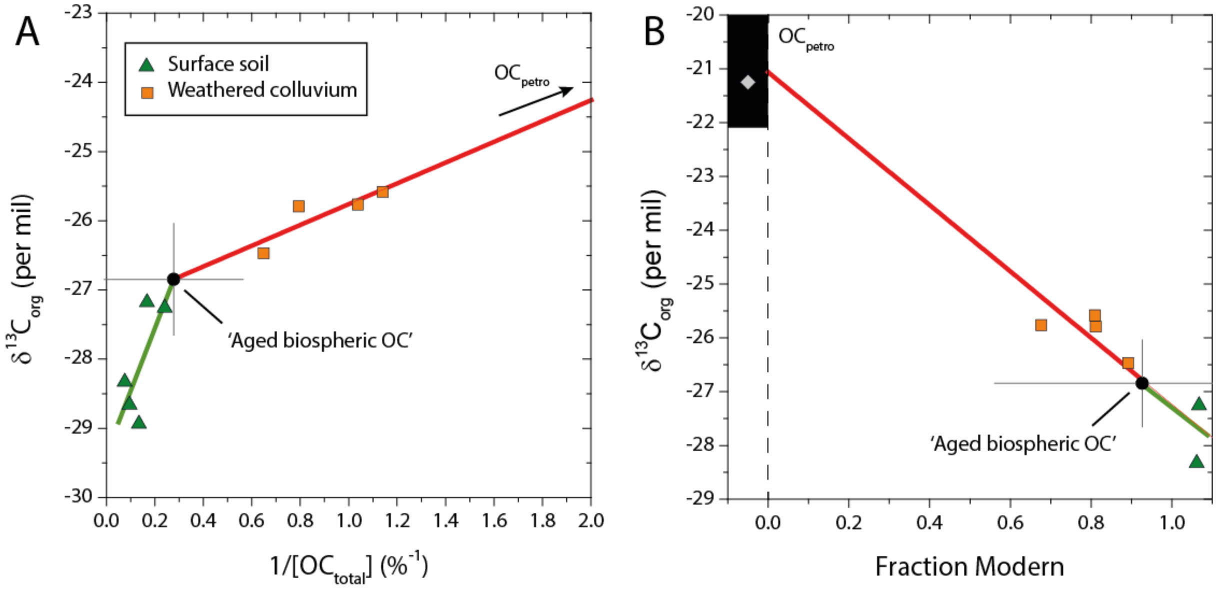

fig. S1. Weathered colluvium from the western Southern Alps. A. The inverse of organic carbon concentration ($1/[OC_{total}]$) versus the stable isotopic composition of organic carbon ($\delta^{13}C_{org}$) for bulk surface soils (triangles) and weathered colluvium (squares). Green line shows linear trend through surface soils ($r^2 = 0.38$). Red line shows linear trend ($r^2 = 0.62$) through weathered materials. The intersection of these linear trends suggests the presence of aged biospheric OC in the weathered colluvium, mixing with OC_{petro}. **B.** The radiocarbon activity of samples reported as the Fraction Modern versus $\delta^{13}C_{org}$ (as per A). Black box indicates range of OC_{petro} composition based on published measurements (*22*) and grey diamond is a river bed material sample downstream of the sampling site. Linear trend through samples ($r^2 = 0.73$) suggests mixing of biospheric OC and OC_{petro}. The aged biospheric end member used in the mixing analysis is shown as a black circle.

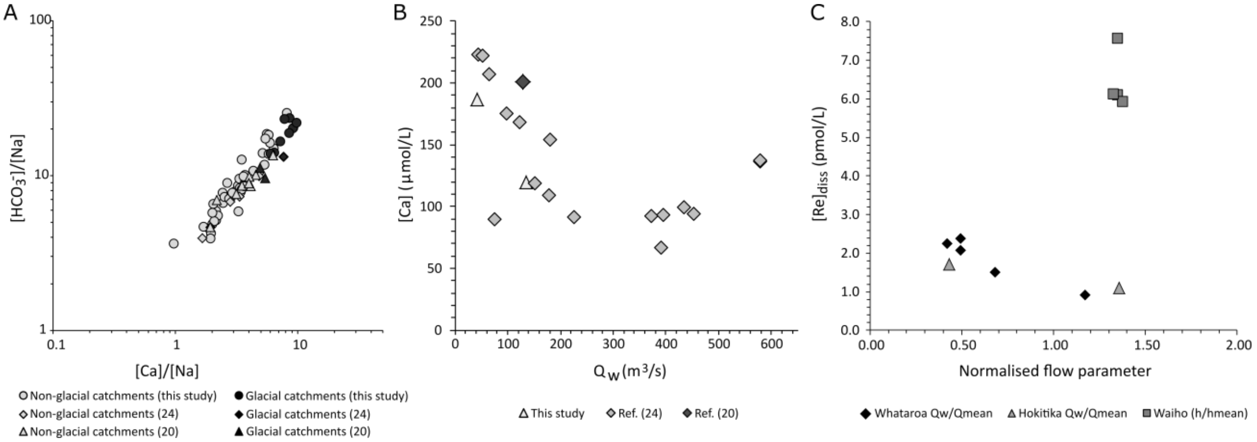

fig. S2. Dissolved major ion concentrations in the western Southern Alps. A. Positive correlation between HCO_3^-/Na and Ca/Na reflects the congruent release of bicarbonate and calcium during chemical weathering. The ion concentrations in the watersheds of the Southern Alps from this study (circles) are shown relative to published data from the Southern Alps (diamonds and triangles (*20*, *24*)). The patterns reflect increased rates of carbonate weathering reactions in the western Southern Alps; this is particularly true for watersheds hosting valley glaciers (black symbols). Note the consistency between the new and published data. Units are mol:mol. **B.** Daily water discharge at time of sampling (Q_w, m^3 s^{-1}) versus [Ca^{2+}] for the Hokitika River for samples from this study (2014, triangles) alongside published data (grey diamonds) from 1999-2000 (*20*, *24*). **C**. River flow versus $[Re]_{diss}$ in the Hokitika, Whataroa and Waiho watersheds where active stream gauge data is available for 2014. The daily water discharge (m^3/s) on sample collection days (Q_w) for the Hokitika and Whataroa watersheds has been normalized to the long-term mean average flow (Q_{mean}) from 1971–2015. An inverse relationship between Q_w/Q_{mean} and $[Re]_{diss}$ reflects a dilution trend. For the Waiho, stage height (h) is normalized to the long-term mean stage height (h_{mean}) from 1983–2015 and did not co-vary during the sampling period.

table S1. River bed materials.

Sample ID	Watershed	Date	Latitude (deg S)	Longitude (deg E)	$[Re]_{BM}$ (ppt)	$[OC]_{BM}$ %	$\delta^{13}C$ (‰)
NZ14-15	Hokitika	14/09/2014	42.89427	171.13438	104.9	0.08	-25.2
NZ14-17	Hokitika	14/09/2014	42.88324	171.15501	51.3	0.09	-22.2
NZ14-19	Hokitika	14/09/2014	42.95557	171.01666	87.2	0.11	-24.1
NZ14-21	Hokitika	14/09/2014	42.74549	171.00066	71.6	0.17	-23.3
NZ14-78	Hokitika	26/09/2014	44.23183	169.23164	109.9	0.17	-22.9
NZ14-80	Hokitika	26/09/2014	42.74551	171.00058	73.4	0.12	-23.5
NZ14-23	Wanganui	15/09/2014	43.16322	170.62808	77.8	0.08	-23.6
NZ14-25	Wanganui	15/09/2014	43.03586	170.44994	53.1	0.08	-24.6
NZ14-27	Poerua	15/09/2014	43.20627	170.521	94.4	0.10	-21.1
NZ14-29	Poerua	15/09/2014	43.13449	170.48598	107.9	0.14	-20.8
NZ14-38	Poerua	16/09/2014	43.15672	170.50438	81.1	0.17	-20.5
NZ14-31	Whataroa	15/09/2014	43.29351	170.41246	236.2	0.10	-24.3
NZ14-33	Whataroa	15/09/2014	43.16091	170.3768	47.4	0.14	-23.2
NZ14-42	Whataroa	16/09/2014	43.29319	170.41313	76.9	0.12	-23.3
NZ14-35	Waitangitaona	16/09/2014	43.28241	170.30812	251.5	0.18	-20.8
NZ14-49	Callery	16/09/2014	43.39675	170.18344	66.2	0.08	-25.7
NZ14-45	Waiho	16/09/2014	43.44185	170.17308	177.4	0.10	-23.1
NZ14-92	Waiho	27/09/2014	43.41808	170.18065	103.7	0.11	-23.0
NZ14-88	Waikupa	27/09/2014	43.44046	170.07457	125.2	0.16	-20.2
NZ14-90	Docherty Creek	27/09/2014	43.38486	170.13333	111.9	0.21	-21.2
NZ14-64	Fox	18/09/2014	43.49958	170.05521	241.2	0.13	-20.7
NZ14-65	Fox	18/09/2014	43.49958	170.05521	172.1	0.13	-20.6
NZ14-67	Fox	19/09/2014	43.48704	170.02962	235.7	0.19	-20.3
NZ14-70	Cook	19/09/2014	43.49912	169.9653	113.9	0.18	-21.7
NZ14-72	Karangarua	19/09/2014	43.57515	169.8051	76.1	0.17	-22.8
NZ14-101	Karangarua	30/09/2014	43.63503	169.95711	74.5	0.11	-23.1
NZ14-104	Karangarua	01/10/2014	43.62964	169.94629	190.8	0.18	-20.7

NZ14-106	Karangarua	01/10/2014	43.61212	169.89484	132.1	0.16	-22.2
NZ14-96	Makawhio (Jacob's River)	29/09/2014	43.57327	169.67892	93.2	0.14	-21.7
NZ14-74	Paringa	19/09/2014	43.71198	169.49315	99.2	0.11	-23.4
NZ14-76	Haast	19/09/2014	43.85398	169.05496	116.7	0.13	-24.3
NZ14-115	Hooker Glacier	04/10/2014	44.03936	169.37926	72.5	0.06	-27.0
NZ14-119	Tasman River	04/10/2014	43.70715	170.17097	82.1	0.06	-26.9
NZ14-121	Jollie River	05/10/2014	43.86295	170.1756	67.6	0.06	-25.9

table S2. Re and OC_{petro} in weathered collvium.

[a]Radiocarbon activity as Fraction Modern. [b]fraction of organic carbon present as petrogenic organic carbon, determined from mixing analysis (see Materials and Methods). [c]petrogenic organic carbon concentration. *f_{petro} assumed to be 1 based on the % [OC] and the stable isotope composition of river bed materials in New Zealand, which are similar to those measured in bedrocks of the mountain belt. **f_{petro} assumed to be 1 in the absence of F_{mod} measurements (based on sampling methodology and type). nd = not determined.

Site	Sample ID	Lat. (ºS)	Long. (ºE)	Sample type	Ref.	$[OC_{total}]$ %	$\delta^{13}C_{org}$ (per mil)	F_{mod} [a]	f_{petro} [b]	UCIAMS Publication Code	$[OC_{petro}]$ % [c]	[Re] (ppt)
Alex Knob, Doherty Creek, New Zealand	NZ14-54	43.40796	170.16727	Weathered colluvium	this study	1.26	-25.8	0.81 ± 0.001	0.13 ± 0.05	178308	0.16 ± 0.06	36.1 ± 0.3
	NZ14-55	43.40602	170.16171	Weathered colluvium	this study	1.54	-26.5	0.89 ± 0.001	0.05 ± 0.02	178310	0.07 ± 0.03	31.4 ± 0.2
	NZ14-56	43.40602	170.16171	Weathered colluvium	this study	0.88	-25.6	0.81 ± 0.001	0.13 ± 0.05	178311	0.11 ± 0.04	23.7 ± 0.2
	NZ14-59	43.40596	170.16115	Weathered colluvium	this study	0.96	-25.8	0.68 ± 0.001	0.26 ± 0.10	178313	0.25 ± 0.10	24.2 ± 0.2
	Average of weathered colluvium (n = 4)										*0.15*	*28.8*
	NZ14-57	43.40596	170.16115	Surface soil	this study	4.16	-27.3	1.07 ± 0.002	nd	178312		24.7 ± 0.2
	NZ14-58	43.40596	170.16115	Surface soil	this study	5.97	-27.2	nd	nd			21.3 ± 0.2
	NZ14-60	43.40596	170.16115	Surface soil	this study	13.24	-28.3	1.06 ± 0.002	nd	178314		52.0 ± 0.4
	NZ14-61	43.40909	170.16335	Surface soil	this study	10.48	-28.7	nd	nd			27.0 ± 0.2
	NZ14-62	43.41448	170.15887	Surface soil	this study	7.44	-28.9	nd	nd			21.7 ± 0.2
	NZ14-90	43.38486	170.13333	Local river bed material	this study	0.21	-21.2	nd	1*			111 ± 1
Ohio Shale, US	244			Weathered rock	(*30*)	1.75	nd	nd	1**		1.75	890
	245			Weathered rock	(*30*)	1.46	nd	nd	1**		1.46	850
	246			Weathered rock	(*30*)	4.29	nd	nd	1**		4.29	17700
	Average of weathered rock (n = 3)										*2.50*	*6480*
	247			Rock	(*30*)	7.30	nd	nd	1**		7.30	89400
	248			Rock	(*30*)	5.95	nd	nd	1**		5.95	115600
	249			Rock	(*30*)	7.13	nd	nd	1**		7.13	74500
	250			Rock	(*30*)	7.80	nd	nd	1**		7.80	81100
	251			Rock	(*30*)	6.75	nd	nd	1**		6.75	78000
	Average of rock (n = 5)										*6.99*	*87720*

table S3. Major and Re concentration data for water samples from the Southern Alps, New Zealand.

Region	Watershed	Collection date	Sample ID	Latitude (degrees S)	Longitude (degrees E)	pH	T	Na^+ (µmol L^{-1})	K^+ (µmol L^{-1})	Mg^{2+} (µmol L^{-1})	Ca^{2+} (µmol L^{-1})	F^- (µmol L^{-1})	Cl^- (µmol L^{-1})	SO_4^{2-} (µmol L^{-1})	$[Re]_{diss}$ (pmol L^{-1})	Total Alkalinity (µmol L^{-1})	HCO_3^- (µmol/L^{-1})
Western Southern Alps	Hokitika	14/09/2014	NZ14-14	42.89427	171.13238	8.52	8.8	66.09	15.13	11.93	162.25	1.05	35.83	25.88	1.91	448.74	441.23
		14/09/2014	NZ14-16	42.88324	171.15501	8.52	11.2	101.30	29.49	17.70	216.75	2.11	47.11	34.30	2.26	613.55	603.33
		14/09/2014	NZ14-18	42.95557	171.01666	8.36	8.4	57.83	25.38	13.99	186.50	1.58	31.31	32.74	1.70	506.08	500.14
		14/09/2014	NZ14-20	42.74549	171.00066	8.2	11.3	83.48	32.05	22.22	178.25	1.58	53.88	29.62	1.54	435.44	431.71
		26/09/2014	NZ14-77	44.23183	169.23164	8.31	8.3	47.39	20.00	9.47	119.25	0.53	34.70	21.83	1.10	349.79	346.01
		26/09/2014	NZ14-79	42.74551	171.00058	8.06	9.6	67.83	25.38	14.40	135.75	1.05	46.83	22.14	1.11	394.47	392.02
	Wanganui	15/09/2014	NZ14-22	43.16322	170.62808	8.14	8.9	259.57	37.18	11.52	251.50	4.21	217.21	74.84	2.88	953.97	947.28
		15/09/2014	NZ14-24	43.03586	170.44994	8.34	9.9	116.52	51.28	23.05	352.50	2.11	67.14	67.66	2.53	929.75	919.50
		16/09/2014	NZ14-40	43.15522	170.62608	8.14	6.4	53.91	19.49	7.00	130.25	1.05	59.52	41.47	1.69	423.24	420.21
		26/09/2014	NZ14-81	43.15533	170.62608	7.98	8.3	56.09	23.85	7.82	159.50	0.53	40.34	30.25	1.50	409.83	407.74
	Poerua	15/09/2014	NZ14-26	43.20627	170.521	8.43	7.5	31.74	42.82	9.47	257.75	1.05	28.21	89.18	3.73	817.79	806.89
		15/09/2014	NZ14-28	43.13449	170.48598	8.21	9.9	46.96	50.51	12.76	263.00	1.05	43.44	80.14	3.38	882.78	875.46
		16/09/2014	NZ14-37	43.15672	170.50438	7.74	8	71.74	31.79	13.58	139.50	0.53	85.19	31.49	1.47	306.17	305.26
		26/09/2014	NZ14-82	43.15686	170.50215	7.92	8.8	46.09	32.56	9.05	132.00	0.53	44.85	25.88	1.14	347.07	345.50
	Whataroa	15/09/2014	NZ14-30	43.29351	170.41246	8.02	8.4	80.43	20.51	13.99	304.50	1.58	34.70	63.61	2.07	724.52	720.62
		15/09/2014	NZ14-32	43.16091	10.3768	7.73	10.7	118.26	49.49	33.74	513.25	2.11	60.37	58.93	2.37	1279.62	1276.09
		16/09/2014	NZ14-41	43.28552	170.40111	8.21	6.4	60.00	15.13	9.88	204.00	1.05	36.39	35.86	1.51	478.83	474.85
		26/09/2014	NZ14-83	43.28552	170.40121	8.04	7.5	43.04	13.59	7.00	150.00	0.53	29.34	24.63	0.90	352.54	350.47
		02/10/2014	NZ14-110	43.28548	170.40146	8.25	8.4	80.87	21.03	14.81	298.00	1.58	35.54	60.18	2.23	740.78	734.09
	Waitangitaona	16/09/2014	NZ14-34	43.28241	170.30812	8.2	7	58.26	30.00	14.81	129.75	1.05	50.21	31.81	1.33	326.60	323.85
		26/09/2014	NZ14-84	43.2822	170.30545	7.86	8	41.30	23.08	8.64	80.00	0.53	37.52	14.97	0.81	163.05	162.35
	Waiho	16/09/2014	NZ14-44	43.44185	170.17308	8.43	2	37.83	63.59	33.33	323.75	1.05	23.41	193.02	6.11	907.41	895.67
		16/09/2014	NZ14-47	43.41813	170.1806	8.48	3.8	53.48	88.97	52.26	493.50	1.05	26.80	192.08	7.56	1111.19	1095.06
		27/09/2014	NZ14-91	43.41808	170.18065	8.26	4.4	45.65	90.51	48.97	446.00	0.53	22.85	165.89	5.94	1017.08	1008.03
		02/10/2014	NZ14-107	43.61212	169.85655	8.34	4.1	57.83	88.72	55.56	491.50	1.05	21.16	198.63	6.13	1108.85	1097.08
	Callery	16/09/2014	NZ14-48	43.39675	170.18344	8.43	5.4	74.78	35.90	22.22	398.00	1.05	26.23	94.79	2.55	893.73	881.98

		27/09/2014	NZ14-93	43.39678	170.18346	8.35	6.9	45.65	24.87	13.99	264.75	1.05	25.11	90.12	2.59	851.34	841.88
		02/10/2014	NZ14-108	43.39725	170.18408	8.42	6.8	58.70	29.74	17.28	317.75	1.05	24.82	115.06	3.09	1032.27	1018.96
	Waiho/ Callery confluence	02/10/2014	NZ14-109	43.3933	170.18042	8.43	6.5	77.39	52.56	32.51	447.75	1.05	26.23	134.39	4.55	1085.42	1071.15
		27/09/2014	NZ14-87	43.44046	170.07457	8.29	9.4	71.30	68.97	29.63	368.75	2.11	52.75	131.90	4.89	1011.23	1001.30
		27/09/2014	NZ14-89	43.38486	170.13333	8.05	9.5	63.04	28.21	20.99	128.25	1.05	51.90	21.52	1.46	418.76	416.23
	Fox	18/09/2014	NZ14-63	43.49958	170.05521	8.62	1.1	56.52	105.13	58.85	441.00	1.05	20.59	266.29	10.67	1344.86	1318.48
		18/09/2014	NZ14-66	43.48704	170.02962	8.52	3.3	112.61	144.10	92.18	722.75	1.58	32.16	274.09	11.55	1620.05	1594.56
		19/09/2014	NZ14-68	43.48704	170.02956	8.94	4.2	80.87	121.28	58.02	482.00	1.05	44.85	148.74	6.26	1178.01	1130.72
		27/09/2014	NZ14-85	43.48598	170.02983	8.31	3.6	76.52	115.64	63.79	549.25	1.05	27.64	190.83	7.68	1289.69	1276.97
		27/09/2014	NZ14-86			8.67	1.1	50.87	111.79	57.20	463.00	0.53	19.18	178.98	6.78	1058.14	1034.81
	Cook	19/09/2014	NZ14-69	43.49912	169.9653	8.48	6.4	51.74	28.46	16.87	174.25	0.53	47.95	41.78	1.85	437.93	431.27
		29/09/2014	NZ14-98	43.49902	169.96536	8.31	7.8	57.83	51.03	32.92	369.25	1.05	28.49	92.92	3.88	881.77	872.77
	Karangarua	19/09/2014	NZ14-71	43.57515	169.8051	8.18	7.3	45.65	19.49	10.29	91.00	0.00	41.75	16.84	0.84	224.44	222.56
		29/09/2014	NZ14-97	43.57513	169.8051	8.05	9.2	49.13	28.21	17.28	171.25	1.05	31.31	52.70	2.28	627.92	624.25
		30/09/2014	NZ14-100	43.63503	169.95711	8.02	9.1	53.91	25.13	32.92	319.75	1.05	20.87	64.86	2.97	885.29	880.54
		01/10/2014	NZ14-103	43.62964	169.94629	7.88	6.6	45.65	38.97	17.28	121.25	0.53	26.80	32.43	1.86	411.52	409.89
		01/10/2014	NZ14-105	43.61212	169.89484	8.05	8.1	77.39	36.41	28.40	257.00	1.05	30.18	57.37	2.84	743.74	739.47
		03/10/2014	NZ14-111	43.57394	169.80774	7.99	7	47.83	24.36	15.23	139.25	0.53	30.47	28.69	1.30	375.08	373.14
	Paringa	19/09/2014	NZ14-73	43.71198	169.49315	7.95	8	43.91	21.54	8.23	91.25	0.53	41.18	16.53	0.93	224.44	223.31
		29/09/2014	NZ14-94	43.71133	169.49055	8.14	9.2	56.09	35.90	16.05	183.75	1.05	35.83	35.55	1.57	333.51	331.00
	Makawhio (Jacob's river)	29/09/2014	NZ14-95	43.57327	169.67892	7.71	9.5	56.52	34.36	17.70	155.50	1.05	38.65	34.92	1.91	401.82	400.71
		03/10/2014	NZ14-112	43.57354	169.67828	7.5	7.2	38.70	17.18	8.23	65.50				0.98	181.56	181.26
		19/09/2014	NZ14-75	43.85398	169.05496	8.09	8.6	59.57	21.54	20.99	220.50	1.05	37.80	34.30	2.01	605.68	601.82
		03/10/2014	NZ14-113	43.85398	169.05486	8	8.4	57.39	19.74	19.75	205.75	1.05	34.98	32.74	1.45	570.51	567.54
		03/10/2014	NZ14-114	44.03936	169.37927	8.02	7.3	46.52	7.44	18.93	169.50	1.05	17.49	27.44	1.80	402.20	399.98
	Hydrothermal	30/09/2014	NZ14-102	43.62964	169.94629	7.02	55.5	17508.70	712.82	184.36	742.25	48.42	4761.64	0.94	0.11	22415.40	22398.17
	Rainwater	15/09/2014	NZ14-RW1	43.389672	170.183759			55.65	3.08	4.94	5.50				0.16		
	Rainwater	02/10/2014	NZ14-RW2	43.389672	170.183759			60.00	3.08	4.53	2.75				0.15		
Eastern Southern Alps	Hooker	04/10/2014	NZ14-116	43.69284	170.09869	8.39	2.1	30.87	11.28	16.46	169.25	0.53	8.74	74.21	0.91	524.72	518.39

Hooker	04/10/2014	NZ14-117	43.69269	170.09903	8.29	4.1	48.26	15.64	28.81	279.75	0.53	11.00	72.03	1.24	655.22	648.90
Tasman	04/10/2014	NZ14-118	43.70715	170.17097	8.43	3.2	67.39	17.44	15.23	240.75	0.53	7.33	82.32	1.05	547.35	540.10
Jollie	05/10/2014	NZ14-120	43.86295	170.1756	8.24	8.4	58.70	7.44	14.40	129.75	1.05	9.31	27.44	0.86	356.91	353.61
Hooker, Jollie and Tasman confluence	05/10/2014	NZ14-122	43.99801	170.19449	8.31	7.7	42.61	9.49	10.29	129.50	1.05	8.74	45.53	1.05	482.13	477.07

table S4. Western Southern Alps watershed average data and dissolved Re yield estimates.

*Glaciers identified from the World Glacier Inventory (*23*). **from Hicks et al., 2011 (*41*) & Jacobson and Blum, 2003 (*20*). ***Determined using Equation 1.

Watershed	Drainage area (km^2)	Average $[Re]_{diss}$ (pmol L^{-1})	Number glaciers*	Total glacier area* (km^2)	Glacier coverage in watershed* (%)	Water discharge (g yr^{-1})**	$[Re]_{BM}/[OC]_{BM}$ (g g^{-1})	OC_{petro} oxidation rate (tC km^{-2} yr^{-1})***
Hokitika	349	1.60 ± 0.37	65	15.2	4.4	2.3E+15	7.3E-08 ± 2.4E-08	15 $^{+7}/_{-5}$
Wanganui	344	2.15 ± 0.66	73	45.4	13.2			
Poerua	136	2.43 ± 1.31	7	3.1	2.3			
Whataroa	454	1.81 ± 0.54	139	44.2	9.7	3.6E+15	1.1E-07 ± 6.1E-08	14 $^{+9}/_{-5}$
Waitangitaona	72	1.07 ± 0.52	12	2.4	3.4			
Callery	95	2.75 ± 0.35	21	23.6	25.0			
Waiho	67	6.43 ± 0.76	9	38.6	57.6	4.0E+14	1.3E-07 ± 7.7E-08	30 $^{+20}/_{-11}$
Fox	92	8.59 ± 2.13	10	45.2	48.9			
Cook	131	2.87 ± 2.03	17	22.1	16.9			
Karangarua	363	2.02 ± 0.69	84	39.0	10.7			
Makawhio	129	1.25 ± 0.65	23	2.6	2.0			
Paringa	226	1.44 ± 0.93	18	10.7	4.7			
Haast	1312	1.75 ± 0.33	226	62.3	4.7	9.7E+15	9.0E-08 ± 3.8E-08	15 $^{+8}/_{-5}$

table S5. Hydrological data for watersheds with river gauging stations.

*Values for the Waiho watershed refer to the stage height and are reported in units of meters.

Watershed	Average Q_w 1971-2015 (m^3/s)	Average Q_w Sept 2014 (m^3/s)	Average Q_w 2014 (m^3/s)	Q_w on days of collection (m^3/s)	Date of collection	Daily discharge (Q_w) / Mean discharge (Q_w mean)	[Re] (pmol L^{-1})
Whataroa	129.07	51.49	129.10	63.95	15/09/2014	0.50	2.07
				63.95	15/09/2014	0.50	2.37
				88.06	16/09/2014	0.68	1.51
				151.41	26/09/2014	1.17	0.90
				54.44	02/10/2014	0.42	2.23
Hokitika	99.64	52.39	105.44	42.95	14/09/2014	0.43	1.70
				135.35	26/09/2014	1.36	1.10
Waiho*	4931*	6495*	6850*	6635*	16/09/2014	1.35*	6.11
				6635*	16/09/2014	1.35*	7.56
				6787*	27/09/2014	1.38*	5.94
				6543*	02/10/2014	1.33*	6.13

table S6. Global watershed averaged Re measurements from mountain rivers draining sedimentary rocks.

River	Area (km^2)	Suspended sediment yield (t km^{-2} yr^{-1})	Ref.	Glacier area (%)	Ref.	Average [Re]$_{diss}$ (pmol L^{-1})	n	Ref.	[Re]$_{BM}$ (pmol kg^{-1})	n	Ref.	Runoff (mm yr^{-1})	Ref.	Dissolved Re yield (mol km^{-2} yr^{-1})	Dissolved Re yield (mol km^{-2} yr^{-1}) / [Re]$_{BM}$ (pmol kg^{-1})
Mackenzie	1712738	254	(*44*)	<0.05%	(*62*)	16.20	7	(*27*)	13130	3	This study	179	(*27*)	2.90E-03	2.21E-07
Taimarli	190	2105	(*43*)	<0.05%	(*23*)	5.63	1	(*9*)	3965	3	(*9*)	2300	(*43*)	1.30E-02	3.27E-06
Upper Chenyoulan	205	2927	(*43*)	<0.05%	(*23*)	4.69	1	(*9*)	3965	3	(*9*)	2000	(*43*)	9.38E-03	2.37E-06
Waipaoa	1570	6797	(*41*)	<0.05%	(*23*)	31.31	2	This study	9044	1	This study	697	(*41*)	2.18E-02	2.41E-06
Chenyoulan	367	8719	(*43*)	<0.05%	(*23*)	8.70	3	(*9*)	3965	3	(*9*)	1900	(*43*)	1.65E-02	4.17E-06
Laonung	853	10785	(*43*)	<0.05%	(*23*)	4.99	1	(*9*)	3965	3	(*9*)	1900	(*43*)	9.47E-03	2.39E-06
Hsiukuluan	249	12851	(*43*)	<0.05%	(*23*)	10.29	1	(*9*)	3965	3	(*9*)	2500	(*43*)	2.57E-02	6.49E-06
Wulu	639	17371	(*43*)	<0.05%	(*23*)	13.86	3	(*9*)	3965	3	(*9*)	2200	(*43*)	3.05E-02	7.69E-06
Yenping	476	19118	(*43*)	<0.05%	(*23*)	12.98	1	(*9*)	3965	3	(*9*)	2100	(*43*)	2.73E-02	6.88E-06
Hualien	1506	20850	(*43*)	<0.05%	(*23*)	19.75	2	(*9*)	3965	3	(*9*)	2100	(*43*)	4.15E-02	1.05E-05
Chihpen	166	21687	(*43*)	<0.05%	(*23*)	7.69	1	(*9*)	3965	3	(*9*)	2400	(*43*)	1.85E-02	4.66E-06
Liwu	435	33103	(*43*)	<0.05%	(*23*)	20.63	6	(*9*)	3965	3	(*9*)	2400	(*43*)	4.95E-02	1.25E-05
Ganga	1033052	519	(*44*)	1	(*63*)	3.90	1	(*27*)	2446	2	(*64*)	477	(*27*)	1.86E-03	7.61E-07
Yukon at Pilot	831400	82	(*45*)	1.1	(*62*)	13.40	7	(*27*)	6353	1	(*65*)	254	(*45*)	3.40E-03	5.35E-07
Yukon at Dawson	264179	125	(*66*)	>1.1	(*62*)	16.86	1	This study	5741	1	This study	259	(*45*)	4.36E-03	7.59E-07
Jollie	140	476	(*41*)	1.7	(*23*)	0.86	1	This study	363	1	This study	2285	(*41*)	1.95E-03	5.39E-06
Brahmaputra	595000	1034	(*44*)	3.1	(*63*)	4.40	3	(*27*)	2589	2	(*64*)	857	(*27*)	3.77E-03	1.46E-06
Hokitika	349	5918	(*41*)	4.4	(*23*)	1.60	6	This study	446	6	This study	6535	(*41*)	1.05E-02	2.35E-05
Haast	1312	4072	(*41*)	4.7	(*23*)	1.75	3	This study	627	1	This study	7412	(*41*)	1.30E-02	2.07E-05
Whataroa	454	10136	(*41*)	9.7	(*23*)	1.81	5	This study	645	3	This study	7923	(*41*)	1.44E-02	2.23E-05
Hooker	107	2596	(*41*)	41.4	(*23*)	1.24	1	This study	415	2	This study	8111	(*41*)	1.00E-02	2.42E-05
Waiho	67	5300	(*20*)	57.6	(*23*)	6.43	4	This study	755	2	This study	5900	(*20*)	3.80E-02	5.03E-05
